# Development and Validation of an Online Oral Health Education Module for Pakistani Parents Using the ADDIE Model

**DOI:** 10.3390/healthcare14121644

**Published:** 2026-06-10

**Authors:** Ushna Shameen, Elavarasi Kuppusamy, Farinawati Yazid, Haslina Rani, Muneer Gohar Babar, Muhammad Khan Asif

**Affiliations:** 1 Department of Family Oral Health, Faculty of Dentistry, Universiti Kebangsaan Malaysia, Kuala Lumpur 50300, Malaysia; p136557@siswa.ukm.edu.my (U.S.); drfarinawati@ukm.edu.my (F.Y.); hr@ukm.edu.my (H.R.); 2 Clinical Oral Health Sciences, School of Dentistry, IMU University, Kuala Lumpur 57000, Malaysia; muneer_babar@imu.edu.my; 3 Department of Oral and Maxillofacial Radiology, Faculty of Dentistry, MAHSA University, Jenjarom 42610, Malaysia; muhammad.khan.scd@stmu.edu.pk; 4 Department of Forensic Odontology and Research, Shifa College of Dentistry, Shifa Tameer-e-Millat University, Islamabad 44000, Pakistan

**Keywords:** oral health education, parents, social media, Facebook, module development, ADDIE model

## Abstract

Background: Parents play a pivotal role in influencing children’s oral health; thus, effective oral health education (OHE) is essential to equip them with the knowledge needed to support their children’s oral health care. In countries such as Pakistan, apart from limited access to dental care and socioeconomic barriers, the widespread lack of OHE is also an important factor contributing to the high prevalence of oral diseases. Conventional OHE approaches are often limited by passive delivery, lack of tailored content and poor accessibility. Social media platforms such as Facebook offer an accessible platform for health education; however, structured, validated, and tailored content is required. Aim: This study aims to develop and validate an online OHE module for Pakistani parents using the ADDIE instructional design model. Materials and Methods: The study was conducted in two phases using the ADDIE model. Parental OHE needs were identified through a questionnaire. An Urdu-language module was developed based on these needs and expert recommendations. Content validation was performed by six experts, followed by face validation with 15 parents. Results: Needs assessment guided the development of a culturally appropriate module covering six main topics. Item-level Content Validity Index ranged from 0.83 to 1.00, with a Scale-level Content Validity Index of 0.94 and a Scale-level Face Validity Index of 0.97. Conclusions: The developed Facebook-delivered OHE module demonstrated high content and face validity and may serve as an accessible and practical strategy for improving parental OHE. Further studies are required to evaluate its effectiveness in improving oral health-related behaviours and outcomes.

## 1. Introduction

Dental caries is a prevalent chronic disease that adversely affects children’s oral health, growth, development, and quality of life [1]. Globally, oral diseases affect approximately 3.5 billion people, with approximately 514 million children experiencing caries in their primary teeth [2]. Despite being largely preventable, dental caries remains highly prevalent in many low and middle-income countries because of inadequate oral health awareness, limited access to preventive services, and delayed treatment-seeking behaviours.

Parents play a crucial role in protecting and promoting the oral health of their children [3]. Their attitudes influence vital parenting practices, including toothbrushing habits and diet, which in turn lead to the development of lifelong oral health behaviours in children [4]. In a study by Ashar et al. (2021), only 32% of parents in Pakistan knew the proper brushing techniques while approximately half were aware of the harms of sugary foods [5]. Thus, OHE for parents is essential to ensure they are equipped with adequate knowledge regarding effective oral health care for their children.

Although OHE is considered one of the most effective ways to raise awareness, traditional approaches such as verbal sessions, pamphlets, and lectures have demonstrated limited long-term change due to low literacy, financial constraints, and restricted access to dental care [6]. These conventional strategies are resource-intensive and often demonstrate limited sustainability in maintaining behaviour change. Conversely, social media has emerged as a popular online medium globally and in Pakistan to provide accessible and cost-effective health information [7]. Social media has become a key source for seeking and sharing oral health information, enabling users to follow updates, share experiences, and join health-related groups at low cost [8]. Despite these advantages, social media content is often variable and unreliable [9]. This gap could be addressed by providing evidence-based, culturally adapted oral health information to ensure the quality, accuracy, and reliability of the information.

In Pakistan, Facebook (66%) remains the most widely used social media platform, followed by YouTube (12%), Instagram (10%), and Twitter (1.7%) [10]. Hence, incorporating Facebook may provide a convenient, affordable, and engaging channel for delivering oral health education to parents.

The ADDIE model is a well-established instructional design framework that provides a systematic approach to developing educational interventions. The model comprises five sequential stages: Analysis, Design, Development, Implementation, and Evaluation [11]. By integrating learner needs assessment, evidence-based content development, expert review, and user feedback, the ADDIE framework facilitates the creation of educational materials that are relevant, well-structured, and responsive to the target audience’s needs. Therefore, this study adopted the ADDIE model to develop and validate a culturally appropriate online oral health education module for Pakistani parents to be delivered via Facebook.

## 2. Materials and Methods

This study was conducted in two phases using the Analysis, Design, Development, and Implementation stages of the ADDIE instructional design framework. Although the ADDIE model consists of five phases, the evaluation stage is beyond the scope of the present study and will be addressed in future research.

Phase 1 comprised the analysis stage to identify the OHE requirements of Pakistani parents. Findings from this phase informed the development of an Urdu-language OHE module.

Phase 2 involved the design, development, and implementation stages of the ADDIE model and focused on the development and validation of an OHE module tailored to Pakistani parents.

### 2.1. Phase I: Assessment of Parental Oral Health Education Needs

#### 2.1.1. Sample Population and Recruitment

Parents of schoolchildren aged 3–12 years in Islamabad were included in all phases of the study. Schools from both urban and rural areas of Islamabad were invited to participate. At the school level, a convenience sampling approach was used, with the first two urban and the first two rural schools that agreed to participate included in the study.

Within each participating school, quota sampling was used to recruit 110 eligible parents. This approach ensured an equal number of respondents from each of the four participating schools and balanced contributions from both urban and rural school settings.

Parents were invited via WhatsApp messages distributed by school authorities after approval from the respective schools. Parents who expressed interest and had internet access were recruited and provided with the participant information sheet and consent form. Data collection was conducted using a mixed-mode approach according to participants’ preference. A total of 316 parents completed the questionnaire online through Google Forms, whereas 124 completed paper-based questionnaires. Findings from this phase were subsequently used to guide the development of the OHE module in Phase II.

#### 2.1.2. Sample Size Calculation

The sample size for Phase 1 was calculated using the Krejcie and Morgan formula, which is appropriate for large populations exceeding one million [12]. Based on the population of Islamabad, estimated at 2,363,863 [13], the minimum required sample size was estimated to be 384 participants. To account for potential non-response, incomplete questionnaires, and participant withdrawal, an additional 14.5% was incorporated, resulting in a final target sample size of 440 participants. This ensured that the required sample size would be maintained despite possible data loss during the study.

#### 2.1.3. Questionnaire Development and Validation

A structured questionnaire was developed to assess the OHE needs of Pakistani parents. Questionnaire development was informed by a previous study [14] and relevant literature on parental oral health knowledge, information seeking behaviour and preferences.

The initial questionnaire was developed by one of the authors (U.S.), who is fluent in both English and Urdu. The questionnaire was prepared as a bilingual instrument in English and Urdu to ensure linguistic accessibility among the target population. Forward and backward translation was conducted by two other bilingual experts. The English version was first translated into Urdu (forward translation), and the Urdu version was then independently translated back into English (back translation). The original and back-translated English versions were compared to ensure linguistic accuracy and conceptual equivalence. Any discrepancies were discussed among the experts and resolved through consensus to ensure clarity, cultural appropriateness, and semantic consistency across both versions. The final validated bilingual version was used for data collection. The questionnaire was divided into 3 sections:

**A: Demographic details of the parents and children**

This section assessed parental age, gender, educational level, household income, number of children, and children’s dental problems.

**B: Oral health status and behaviour of the children**

Sample items included frequency of toothbrushing, use of fluoridated toothpaste, parental supervision during brushing, sugary food consumption, and regular dental visits.

**C: Oral health education (OHE) needs**

This section explored parents’ motivation for seeking oral health information, preferred and trusted information sources, exposure to oral health information through social media and preferred OHE formats and topics.

In addition to the questionnaire development process, further expert input was sought to strengthen the content validity and scope of the instrument. A panel of paediatric dentistry and dental public health experts reviewed the preliminary results and provided input on priority content areas, prevalent misconceptions, and clinically relevant topics in children’s oral health.

#### 2.1.4. Pretesting of Questionnaire

The newly developed questionnaire was pretested among 40 parents from the selected schools. However, these participants were not included in the main study. Pretesting was conducted to evaluate the clarity, comprehensibility, relevance, sequencing, and acceptability of the questionnaire items, as well as the feasibility of administration.

Participants completed the questionnaire and provided feedback on items that were unclear, difficult to interpret, repetitive, or irrelevant. Completion time and any practical issues encountered during administration were also recorded. The research team analysed the feedback obtained during pretesting and made minor modifications to improve item phrasing, response options, layout, and overall readability. The revised questionnaire was subsequently administered in the main study. However, formal psychometric testing, including reliability analysis (e.g., Cronbach’s alpha), was not conducted at this stage.

### 2.2. Phase II: Development and Validation of an Oral Health Education Module

Phase II consisted of the design, development, and implementation phase of the ADDIE model. The OHE module was created in the Urdu language using simple and non-technical terms and concise sentences to ensure clarity, cultural appropriateness, and accessibility.

#### 2.2.1. Content Development

The OHE module subtopics and topics were adapted from the book “Basic Guide to Oral Health Education and Promotion” by Chapman and Felton [15], supplemented with 10 peer-reviewed articles and five books authored by oral health professionals published between 2013 and 2022. The module consisted of six main topics, as shown in Table 1. Parents’ self-reported OHE needs were used to guide the topic selection for the module. Any discrepancies were discussed among the research team until a consensus was reached. Visually appropriate and culturally relevant stock photographs for each topic were sourced from Freepik. A storyboard was then written using the finalised text and illustrations and submitted to the graphic designer for the development of the OHE module.

#### 2.2.2. Content Materialisation

The module’s content was designed into educational materials, including infographic posters and short informational videos. Graphic design principles, including typography, colour, and layout, were applied in accordance with PEMAT guidelines [16,17] to improve clarity and audience engagement. Final designs, graphics, and voice-overs for videos were produced using Canva Pro and reviewed by the authors.

#### 2.2.3. OHE Module Upload to Facebook

**Figure 1:**
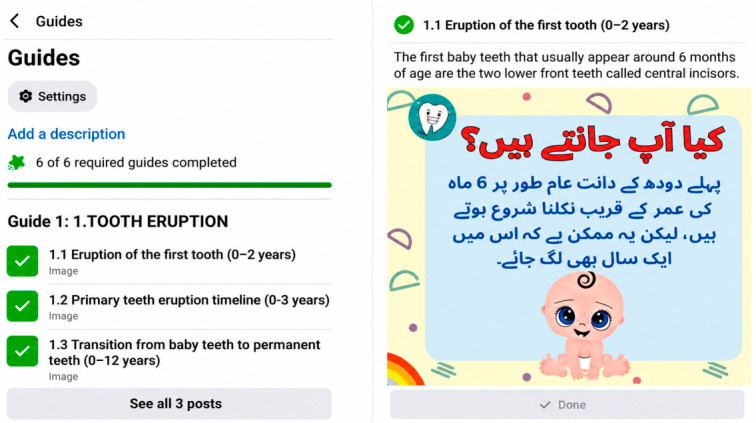
Example of a Facebook group post from the oral health education module. The screenshot illustrates the delivery of educational content through the Facebook group guide entitled “*Tooth Eruption.*” The post shown is titled “کیا آپ جانتے ہیں؟” (Did You Know?) and informs parents that the first primary teeth usually begin to erupt at approximately six months of age, although eruption may be delayed until around one year in some children.

A Facebook group entitled “Oral Health Education for Children” was created to disseminate the OHE materials. Six guides representing the main module topics are presented in Figure 1. Each post included a title, caption, and instructions in English and Urdu. A welcome post guided the participants to navigate the group. Engagement with the module was monitored through self-reported completion indicators (i.e., “I am done” responses). No Facebook analytics, such as views, reach, or interaction metrics, were collected. A sample of post on the first topic ‘Tooth Eruption’ is shown in Figure 1.

#### 2.2.4. Content Validation

An expert panel comprising three pediatric dentists and three dental public health specialists, proficient in both Urdu and English, evaluated the module’s relevance and clarity using a 4-point Likert-scale questionnaire, and the ratings were quantified using the Item-level Content Validity Index (I-CVI), consistent with recommendations suggesting the involvement of six to ten professionals with relevant expertise in the subject area [18,19].

The Item-level Content Validity Index (I-CVI) was calculated for each educational component. Subsequently, the same panel of experts evaluated the overall module using a structured instrument adapted from previously validated studies. The assessment consisted of 19 items distributed across four domains: writing style, structure and presentation, objectives, and overall relevance of the educational materials [20,21].

The module was scored using a 4-point Likert scale reflecting the degree of agreement and content validity. The content validity index was calculated using two metrics: The Item-level Content Validity Index (I-CVI) and the Scale-level Content Validity Index (S-CVI/Ave). A CVI score of ≥0.83 was considered a satisfactory level of content validity [22].

#### 2.2.5. Face Validation

For face validation, 15 parents were purposively selected from the 440 parents who participated in the Phase I needs assessment. Selection was based on parents’ willingness to participate further, availability, and fulfilment of the eligibility criteria, including having an active Facebook account and access to the internet. These criteria were applied because the OHE module was intended to be delivered via Facebook, and participants needed to access and review the module in its intended format. Participants were invited to join the Facebook group to evaluate and rate the OHE module across three domains (12 items) related to writing style, structure and presentation, and motives, using a 4-point Likert scale. Sample size estimation for face validation does not involve statistical power or effect size calculations.

Previous methodological studies indicate that approximately 15–20 participants are generally sufficient for face validation during educational material development [23]. The results were calculated using the Item-level Face Validity Index (I-FVI) and the Scale-level Face Validity Index based on I-FVI (S-FVI/Ave) [24].

## 3. Results

### 3.1. Phase I: Assessment of Parental OHE Needs

A total of 440 parents participated in the needs assessment phase of the study, with ages ranging from 22 to 56 years (mean: 34.8 ± 7.2 years). The sample was predominantly female (64%), with males comprising 36%, indicating that mothers were the primary respondents. There were 220 parents from the urban schools, and the remaining 220 parents were from the schools in rural locations.

The finding showed that only 7.7% of parents deliberately sought oral health information for their children, indicating low intentional information-seeking behaviour. Almost half of the parents reported that their motivation to seek information is often triggered by child-related symptoms (47.1%).

Dentists were the most preferred (70%) and trusted (72.4%) source of oral health information. Pamphlets (7.1%), newspapers (3.9%), and books (0.9%) were less commonly used. Among the social media platforms, Facebook (12.1%) was the most preferred platform, followed by Instagram (11%). However, less than one third of the parents trusted both platforms as sources of oral health information.

However, 88% of parents reported receiving oral health information through social media, reflecting passive exposure to content such as posts, videos, advertisements, and shared messages during routine use of social media. Only half of the parents considered the social media information they have received to be relevant or comprehensive, while others reported it as too general (37%) or insufficient (13%).

More than 90% expressed interest in age-specific oral health information, preferably in both written and video formats. Oral hygiene care (37%) was the most preferred topic, whereas 17.7% showed interest in diet-related caries prevention. The summary of parent’s preferred sources of information, exposure to oral health information, their perception of the information quality, preference for age-appropriate information and the format of OHE materials are shown in Table 2.

### 3.2. Phase II: Development of the OHE Module

#### 3.2.1. Content Design and Layout

**Figure 2:**
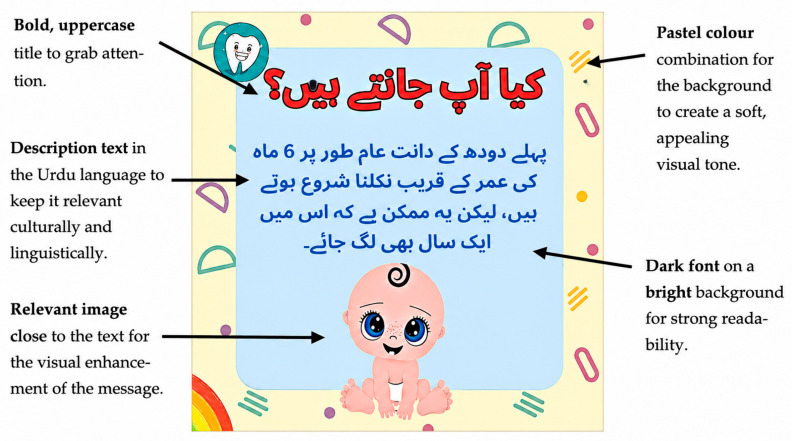
Example of the infographic design and content used in the oral health education module. The title “کیا آپ جانتے ہیں؟” translates to “Did You Know?”. The body text translates to: “The first baby teeth usually begin to erupt at around 6 months of age. However, in some children, eruption may be delayed until approximately 1 year of age.”.

Findings obtained from the parental need assessment, recommendations provided by oral health experts and evidence derived from the relevant literature were used to guide the development of an OHE module. Graphic design elements were incorporated to develop visually appealing and functional OHE materials for parents, as shown in Figure 2.

#### 3.2.2. Content Generation

The module included six main topics and 47 subtopics to ensure comprehensive information for parents. The educational materials were developed for different age groups based on their specific informational needs.

**Figure 3:**
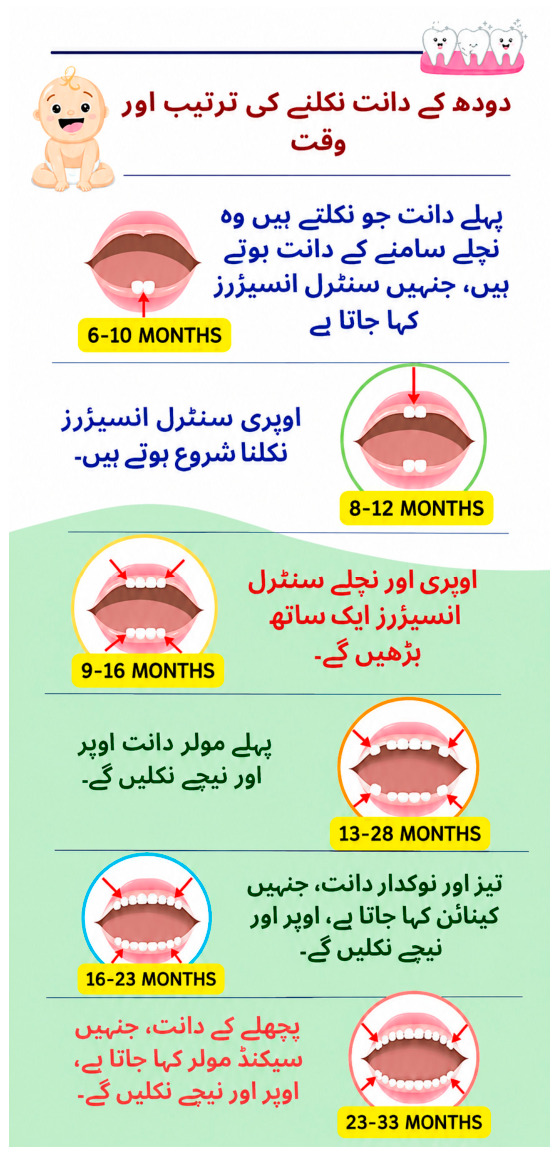
Infographic on the sequence and approximate timing of primary tooth. The Urdu title translates to “Timing and Sequence of Primary Tooth Eruption”. The infographic illustrates the typical eruption sequence of primary teeth and the age ranges at which different groups of primary teeth are expected to emerge.

The module was structured with tooth eruption as the first topic. This topic provided parents with information on the sequence of tooth eruption to enable monitoring during both primary and permanent dentition stages. An infographic on the eruption sequence of deciduous teeth is shown in Figure 3.

**Figure 4:**
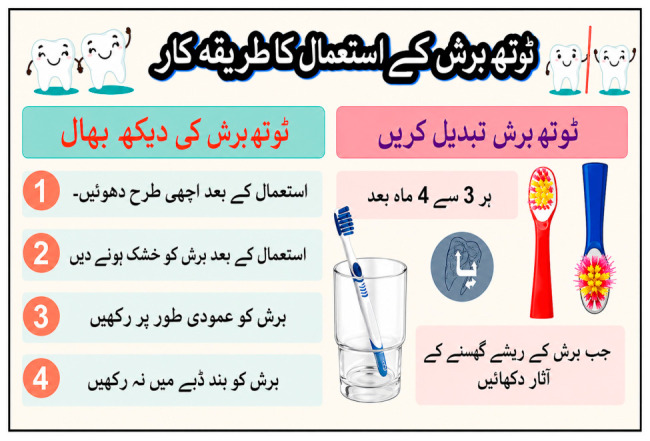
Infographic on oral hygiene care. The Urdu title translates to “How to Use and Care for a Toothbrush?”. The infographic provides guidance on proper toothbrush maintenance, including rinsing the toothbrush thoroughly after use, allowing it to air dry, storing it upright, and avoiding storage in a closed container. It also advises parents to replace the toothbrush every three to four months or when the bristles show visible signs of wear.

The second module topic, “oral hygiene care”, covers oral hygiene practices for infants and children, including available oral hygiene aids, brushing techniques, and strategies to overcome toothbrushing difficulties. An example is illustrated in Figure 4.

**Figure 5:**
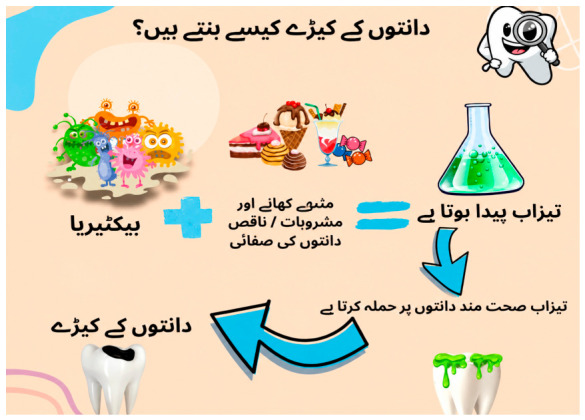
Infographic on Dietary and dietary advice. The Urdu title “دانتوں کے کیڑے کیسے بنتے ہیں؟” translates to “How Do Dental Cavities Form?”. The infographic explains how bacteria, sugary foods or drinks, and poor oral hygiene produce acids that attack teeth and lead to dental caries.

The third topic, “Diet and dietary advice”, provided information related to diet and healthy eating habits for children. The fourth topic focused on dental caries, including its aetiology, signs and symptoms, stages, and complications. It also provided parents with guidance on identifying early visible signs of caries at home to improve awareness and encourage timely dental consultation, rather than promoting self-diagnosis or replacing professional assessment. An example is illustrated in Figure 5. The fifth topic of the module aims to educate parents about professional dental care by highlighting the importance of regular dental visits and fluoride varnish application, and by dispelling prevalent misconceptions through a set of myths and facts about dental caries to help parents distinguish between outdated beliefs and evidence-based practices.

**Figure 6:**
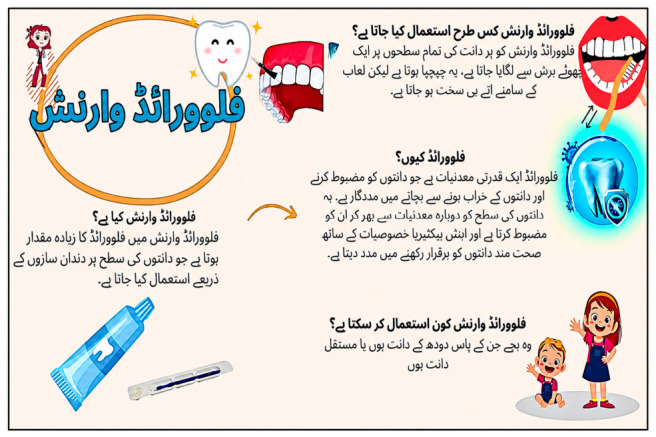
Infographic on the role of fluoride in the prevention of dental caries and maintenance of oral health. The Urdu title “فلورائڈ وارنش” translates to “Fluoride Varnish”. The infographic explains what fluoride varnish is, how it is applied to the tooth surface, and how it helps strengthen teeth and prevent dental caries.

The professional preventive treatments of fluoride varnish and fissure sealants are also introduced to parents, explaining how they help prevent tooth decay in children. This is illustrated in an example in Figure 6. The sixth and last topic in this module focused on educating parents about the early tooth loss of primary teeth.

#### 3.2.3. Module Validation

The panel of six experts first evaluated individual materials, then conducted an overall assessment of the entire module for comprehensive validation. According to the Content Validity Index (CVI) analysis, the Item-level CVI (I-CVI) for all materials ranged from 0.83 to 1, except for one subtopic (suitable drink for your child, age 7–12 years) that received a score of 0.67. This item was revised based on expert feedback to improve clarity and alignment with age-appropriate dietary recommendations before final inclusion in the module. The Scale-level Content Validity Index/Average (S-CVI/Ave) for the OHE materials was 0.97.

The same panel of six experts assessed the entire module based on the level of agreement for four domains. The I-CVI for individual items ranged from 0.83 to 1, and the S-CVI/Ave for the module was 0.94.

#### 3.2.4. Face Validation of the Module by Parents

A total of 15 parents participated in the face validation of the OHE module. The sociodemographic characteristics of participants are given in Table 3. Parents rated the module based on three domains, and the face validity index (FVI) analysis revealed that the S-FVI/Ave was 0.97, as shown in Table 4.

## 4. Discussion

The study aimed to develop and validate an OHE module tailored for Pakistani parents based on their needs using a systematic instructional design of the ADDIE model. The incorporation of the parental need analysis, combined with expert input, ensured a structured development of the module with high content and face validity, indicating its relevance and suitability for the targeted population.

The findings from the needs assessments showed that only a small proportion of parents actively sought oral health information, with symptom-triggered information seeking most common, reflecting a reactive rather than proactive approach to children’s oral health management. Similar patterns have been reported in studies conducted in Malaysia, where parents primarily sought information only after noticing changes in their child’s oral condition [25], and the motivating factors for the majority of the parents to actively seek information on early childhood caries (ECC) include aesthetic concerns or disruptions to daily activity [14].

Traditionally, OHE is delivered primarily by healthcare professionals during clinical visits, which requires regular access to dental services. In Pakistan, recent evidence highlights substantial challenges in access, affordability, and utilisation of dental services, particularly within the public sector. These barriers limit opportunities for parents to receive oral health information through conventional clinician-based interactions [26]. This highlights the need to move beyond traditional delivery models and explore more accessible and affordable platforms. Social media has become a popular source of information, but concerns about content quality and reliability highlight the need for structured and validated education modules [27].

The OHE module was developed in Urdu to ensure cultural relevance and ease of understanding. Parents preferred receiving information in their first language, as it is easy to comprehend [28]. Complex concepts were explained using plain language, visual images, and analogies to facilitate comprehension, consistent with studies demonstrating effectiveness of these approaches [29]. Videos were prepared to explain concepts that are difficult to convey through voice-overs alone. The integration of visual and auditory elements enhances knowledge acquisition and promotes behavioural change [30].

The revised module was shared through a Facebook group, “Oral Health Education for Children,” providing an online community-based OHE platform with interpersonal communication between parents and dentists. Content validation and face validation were incorporated during module development to ensure that the educational materials were relevant, understandable, and appropriate for the intended audience [31].

Six oral health experts participated in content validation. Content validation results demonstrated excellent agreement among experts, indicating that the developed module was appropriate in terms of accuracy, relevance, and clarity. Validation of the module by dental professionals also enhanced its credibility and reliability. Face validity conducted with 15 parents yielded an S-FVI/Ave of 0.97, indicating that the module was perceived as easy to understand, visually attractive and has relevant content [32]. However, no usability testing was conducted in this study. Therefore, conclusions regarding user interaction, engagement behavior, or practical usability cannot be drawn. The evaluation was limited to content and face validity only, which are preliminary steps in educational material development.

Overall, the findings suggest that a structured, culturally appropriate, and evidence-informed OHE module can be successfully developed using the ADDIE framework. However, further studies are required to evaluate its effectiveness in changing parental knowledge, attitudes, and oral health behaviours.

## 5. Limitations of the Study

Several limitations should be considered when interpreting the findings of this study. Participants were recruited from selected schools in Islamabad using non-probability sampling methods. Therefore, parents from other regions of Pakistan, particularly rural and underserved populations, may not be adequately represented. This may limit the generalisability of the findings.

Although face validation was conducted among parents, the sample consisted predominantly of individuals with tertiary-level education. Consequently, the perspectives and comprehension levels of parents with lower educational attainment or limited literacy may not have been adequately represented. Since these groups may benefit most from simplified oral health educational materials, additional validation among more educationally and socioeconomically diverse populations is recommended before wider implementation of the module.

The needs assessment questionnaire was pretested to evaluate clarity, comprehensibility, and feasibility; however, formal psychometric reliability testing was not conducted. The questionnaire was developed mainly to gather exploratory information regarding parents’ motivation for seeking oral health information, preferred and trusted information sources, social media usage, and preferred OHE formats and topics, which were used to guide module development rather than to establish a psychometric scale for outcome measurement. Therefore, emphasis was placed on content relevance and usability instead of scale reliability. However, the lack of reliability assessment remains a limitation of the study.

Finally, the behavioural or clinical outcomes of the OHE module were not evaluated. Therefore, the real-world impact of the module on parental behaviours and children’s oral health status remains unknown.

## 6. Clinical Implications

This study presents a systematically developed and validated OHE module for Pakistani parents that may serve as a useful educational resource. The module is culturally appropriate and designed to address key gaps in parental oral health knowledge identified during the needs assessment phase.

However, because this study did not assess behavioural or clinical outcomes, no conclusions can be drawn about its effectiveness in improving oral health practices. Therefore, the module should be considered as an educational resource with potential application in community and digital health education settings.

## 7. Future Directions

Future studies should focus on evaluating the effectiveness of the module in broader populations, particularly rural areas where digital access and education needs may differ. Future studies should assess the module’s effectiveness using a randomised controlled trial with pre- and post-intervention assessments, such as KAP surveys and clinical evaluations for caries and oral hygiene.

To increase its reach, the module could be translated into other languages and piloted among diverse language groups. Its application can also be tested through various platforms, e.g., Instagram, TikTok, websites, or Mobile apps, to reach different populations.

Integration of AI-powered chatbots may further enhance long-term benefits by providing real-time information to parents and improving access to reliable oral health education [33].

## 8. Conclusions

A culturally tailored OHE module for Pakistani parents was systematically developed and validated using the ADDIE instructional design framework. The module demonstrated strong content and face validity, indicating that it is appropriate in terms of clarity, relevance, and presentation for the target population. However, this study did not assess behavioural or clinical outcomes; therefore, its effectiveness in improving oral health literacy, parental behaviours, or children’s oral health status remains to be established. Further intervention-based studies are required to evaluate its real-world impact.

## Figures and Tables

**Table 1:** Major topics and educational content included in the oral health education (OHE) module for Pakistani parents.

Number	Topic
1	Tooth Eruption
2	Oral hygiene care
3	Diet and dietary advice
4	Dental caries
5	Professional dental care
6	Importance of deciduous teeth

**Table 2:** Parental oral health education (OHE) needs.

Question	*N* (%)
1. Preferred Source	
Dentist	312 (71%)
Friends/Family	104 (24%)
Facebook	53 (12%)
Instagram	48 (11%)
2. Trusted Sources	
Dentist	314 (72%)
Friends and Family	176 (40%)
Facebook	113 (26%)
Instagram	90 (21%)
3. Received information from social media	388 (88%)
4. Perception of information quality	
Relevant	156 (40%)
Too general	145 (37%)
Insufficient	52 (13%)
Comprehensive	39 (10%)
5. Preference for age-appropriate information	406 (92%)
6. Preference for information format	
Both (Written materials & Videos)	248 (56%)
Written materials	122 (28%)
Videos	70 (16%)
7. Oral health topics	
Oral Hygiene Care	142 (32%)
Tooth eruption in children	113 (26%)
Diet for cavity prevention	78 (18%)
Self-assessment of caries	67 (15%)
Causes of cavities	40 (9%)

Note: Participants were allowed to choose multiple options for item 1 and 2; therefore, the final percentage exceeds 100%.

**Table 3:** Summary of parental demographic characteristics.

Demographic Factor	*N* (%)
Gender	
Male	7 (47%)
Female	8 (53%)
Age (years)	
25–29	5 (33%)
30–34	6 (40%)
35–39	3 (20%)
≥40	1 (7%)
Highest Level of Education	
FSC (High School)	2 (13%)
Bachelor’s Degree	9 (60%)
Master’s Degree	4 (28%)

**Table 4:** Face validity assessment of the oral health education module among parents.

Domains and Items	I-FVI
A. Writing Style	
1. The text is clear and easily readable	1
2. The chosen font style and size are appropriate	1
3. The spacing between the texts is appropriate and consistent	0.83
B. Structure and Presentation	
4. The language used is easy to understand	1
5. The layout is clear and attractive	1
6. The scope of content is sufficiently in-depth	0.87
7. Images used easily attract readers	1
8. Images presented aid in understanding the content	1
C. Motives	
9. The content of the module is easy to understand	1
10. The module catches your attention	1
11. The module increases your understanding of dental caries and oral health care for your child	1
12. The module addresses relevant aspects of oral health care measures and dental caries prevention in children	1
S-FVI/Average	0.97

## Data Availability

The data presented in this study are available on request from the corresponding author due to confidentiality and ethical considerations.

## Abbreviations

OHE	Oral Health Education
CVI	Content Validity Index
FVI	Face Validity Index
I-CVI	Item-level Content Validity Index
I-FVI	Item-level Face Validity Index
KAP	Knowledge, attitude and practice
PEMAT	Patient Education Materials Assessment Tool
S-CVI/Ave	Scale-level Content Validity Index based on I-CVI
S-FVI/Ave	Scale-level Face Validity Index based on I-CVI
